# Microwave Sensors for Dielectric Characterization: Planar Architectures, Extraction Methods, and Emerging Applications

**DOI:** 10.3390/s26165312

**Published:** 2026-08-21

**Authors:** Feifei Tan, Changjun Liu

**Affiliations:** 1 College of Communication Engineering, Chengdu University of Information Technology, Chengdu 610225, China; tff926@cuit.edu.cn; 2 College of Electronics and Information Engineering, Sichuan University, Chengdu 610207, China

**Keywords:** microwave sensors, dielectric characterization, planar architectures, complex permittivity, extraction methods, microfluidic sensing, nondestructive evaluation, machine learning

## Abstract

Microwave dielectric characterization is essential for material evaluation, process monitoring, biomedical sensing, and nondestructive testing. This review critically evaluates planar microwave sensors for dielectric characterization, with the core scope restricted to printed microstrip and coplanar-waveguide structures, SRR/CSRR and DGS configurations, substrate-integrated waveguides, interferometric sensors, and microfluidic platforms. Adjacent non-planar or system-level techniques are included only when they provide transferable lessons in calibration, inversion, or deployment. Unlike earlier surveys that primarily catalog devices or extraction methods, the literature is organized here through a design-decision hierarchy linking architecture, operating principle, readout mechanism, sample interface, and application. Representative approaches are compared not only by frequency, sensitivity, Q-factor, and sample volume, but also by calibration burden, fabrication tolerance, environmental robustness, cost, and scalability. Particular attention is given to uncertainty sources in practical measurement chains, FR-4 and PCB manufacturing variability, long-term drift and sensor aging, and the application-specific limitations of machine-learning-assisted inversion. The resulting synthesis provides design-oriented guidance for selecting and translating planar microwave sensors into reliable industrial, biomedical, and microwave-processing measurement systems.

## 1. Introduction

### 1.1. Background and Motivation

Dielectric constant measurement is a basis of modern material characterization in both fundamental scientific research and a wide range of engineering techniques. In industrial situations, accurate permittivity enables quality control of polymers [1], composites [2], and coatings [3], coal [4] supports analysis of mixture composition [5] and provides rapid indicators of moisture content [6] and curing progression in adhesives, resins [7], and cement-like materials [8]. In biomedicine, the dielectric properties of biological tissues and fluids provide label-free contrast mechanisms associated with water content [9], ionic concentration [10], and molecular composition, e.g., breast tissues [11,12]. In public security, dielectric constant measurement enables the identification of hazardous objects through advanced polarimetric decomposition methods in real-time scanners [13]. It facilitates applications ranging from biosensing to diagnostics and the monitoring of microfluidic samples.

Temperature-dependent material permittivity is critical during microwave heating and drying to avoid the so-called thermal runaway [14,15] and to evaluate microwave-radiation effects in biological or agricultural materials [16]. Besides these domains, dielectric constant measurement also plays an important role in food safety and quality, e.g., infection detection [17], evaluation [18], and microwave spectroscopy of food products [19]; microwave circuit design [20] and PCB substrate qualification [21]; and emerging microwave-assisted chemistry [22], where permittivity governs electromagnetic energy coupling and heating generation. Moreover, the dielectric characterization of indoor building materials, such as glass and ceramics, provides foundational data for analyzing radio propagation at THz frequencies [23]. Consequently, there is a continuing demand for nondestructive, rapid, and cost-effective measurement techniques capable of providing reliable dielectric characterization across diverse materials and operational environments. Recent reviews have also summarized microwave sensor platforms, extraction mechanisms, and application trends for permittivity measurement, highlighting the importance of differential signals, metamaterial-inspired sensing, and microfluidic integration [24].

Nondestructive, real-time dielectric constant measurement is particularly valuable because it enables in situ monitoring and closed-loop control [25]. In certain situations, material properties evolve continuously, and destructive sampling is impractical or even impossible.

In manufacturing and quality control, rapid permittivity tracking supports online inspection of polymer curing, adhesive bonding and conductive-adhesive curing [26], coating uniformity, and moisture fluctuations, including grain-moisture monitoring [27]. It reduces waste and improves process repeatability.

In biomedicine and microfluidics, nondestructive measurement preserves precious samples and allows continuous observation of dynamic biochemical reactions, concentration changes, and cell-related processes [28]. Real-time dielectric monitoring is also essential in microwave heating and microwave-assisted chemistry, where dielectric properties directly determine electromagnetic energy absorption, penetration depth, and heating efficiency. Since the permittivity of many materials is strongly temperature-dependent, microwave processing can exhibit nonlinear positive feedback: as a localized region heats up, its permittivity and loss may increase, leading to stronger microwave absorption and further heating, which ultimately leads to hot-spot formation and eventually to thermal runaway. Recent developments include an integrated system based on an oblique aperture ridge waveguide and artificial neural networks, which enables real-time monitoring of complex permittivity during microwave heating to prevent thermal runaway [29]. High-temperature ridge-waveguide systems further extend such monitoring to wide-permittivity materials and elevated temperatures for microwave-assisted metallurgy [30]. Continuous permittivity sensing, therefore, provides a method to detect initial instability and control microwave power, which is essential for safe and reproducible microwave chemical synthesis and microwave heating. Accordingly, nondestructive, real-time dielectric measurement techniques are increasingly sought as enabling tools for both industrial monitoring and microwave-driven biomedical and chemical applications.

Recent theoretical advances have improved the performance of permittivity measurements. For example, a multi-parameter inversion method enables the simultaneous reconstruction of permittivity and conductivity in stratified composite media [31]. Furthermore, a contactless identification algorithm (DIMAR) has been introduced, utilizing a moving RFID system and synthetic aperture radar (SAR) concepts to characterize complex permittivity and conductivity with fine resolution [32]. A machine-learning-aided approach has been developed to reconstruct the complex permittivity of dispersive materials directly from amplitude-only transmission measurements, effectively eliminating the requirement for a vector network analyzer [33]. A method based on machine-learning-optimized databases has been developed to predict the relative permittivity of various ceramics at microwave frequencies, supporting the design of components for next-generation networks [34].

Dielectric spectroscopy is a valuable analytical tool to study the dielectric properties by measuring the polarization response of the materials, which possesses the characteristics of being nondestructive and providing comprehensive information. It can be performed in either the frequency domain or the time domain.

Frequency-domain dielectric spectroscopy (FDS) measures the polarization response under an AC electric field, enabling rapid acquisition of data at various excitation frequencies, either point by point or via frequency sweep. It can provide information about insulation states such as moisture content and aging degree. This method is widely used in the condition assessment of the resin curing process and oil-paper insulation [35,36,37,38]. At present, frequency-domain dielectric spectroscopy (FDS) measurement systems are typically constructed using commercial dielectric spectroscopy instruments, such as the IDAX300 from Megger, the DIRANA from OMICRON, and the Concept 80 from Novocontrol, with frequency ranges spanning from sub-MHz to several GHz. These FDS measurement setups have become increasingly mature; however, they suffer from high procurement costs, long maintenance cycles, and extended measurement times, making them unsuitable for widespread application. Currently, some researchers have been developing dielectric spectroscopy systems. S. Chatterjee et al. employed a linear excitation source as the signal generator and experimentally demonstrated that, while maintaining the accuracy of the dielectric spectrum, the measurement time was reduced by approximately 75.9% compared with conventional frequency-domain dielectric spectroscopy [39]. Michael Jaya et al. proposed sampling only one-tenth of the period of the response signal and reconstructing the full waveform through data fitting. Although this method significantly shortens the measurement time, the results are highly susceptible to the original data, and the accuracy is difficult to guarantee [40].

In contrast, time-domain dielectric spectroscopy (TDS) instruments are more economical and portable than frequency-domain counterparts, as they can be powered by a small battery and deliver broadband information from a single step-pulse response. Therefore, various TDS-based approaches have been explored. Common time-domain reflectometry (TDR) methods obtain the complex dielectric permittivity of a material under test (MUT) by comparing reflected and incident waves [41,42], with subsequent approaches incorporating multiple reflected signals and full waveforms. These methods, however, require knowledge of the source function, which is susceptible to temperature variations. To circumvent the challenge of accurate source function determination and to enable process monitoring under time-varying source function conditions, recent studies have proposed methods that eliminate the need for source function characterization.

With the increasing number of wireless application scenarios, wireless transmission systems have become one of the key supporting technologies in contemporary information society. Based on wireless transmission technology, microwave sensing technology, which features non-physical contact, non-destructive operation, and high real-time performance, has experienced rapid development. Microwave sensing technology primarily enables the detection and measurement of physical quantities such as object shape, material properties, component concentrations in mixtures, and displacement parameters, by monitoring changes in the electromagnetic characteristics of substances. This technology is widely used in fields such as industry, agriculture, healthcare, and biology. Traditional waveguide-based microwave sensors offer advantages such as high quality factor, strong anti-interference capability, low loss, high power handling, and ease of fabrication. However, due to their large size and difficulty in integration, they are rarely used in permittivity detection and are generally applied in metallic crack detection [43].

Planar microwave sensors have emerged as an attractive platform for measuring dielectric constants since they are compact, low-cost, and sensitive to localized near-field interactions. They are usually implemented by standard printed-circuit-board (PCB) technologies such as microstrip or coplanar waveguide (CPW). These sensors are lightweight and easily scalable, enabling deployment in both laboratory and industrial environments. Their planar geometry further facilitates straightforward integration with microfluidic channels, allowing controlled handling of microliter-to-nanoliter liquid samples and enabling lab-on-PCB systems for biochemical analysis, point-of-care testing, and continuous reaction monitoring. Moreover, the sensing mechanism relies on evanescent electromagnetic fields concentrated around resonant gaps, interdigital capacitors, or metamaterial-inspired elements, e.g., SRR/CSRR and DGS, thereby enhancing field confinement and improving sensitivity to permittivity variations. In addition to dielectric characterization, the same SRR field-localization principles have been adapted to related planar sensing functions such as rotation sensing [44]. Compared with conventional waveguides and cavity resonators, planar sensors offer a more compact and cost-effective alternative, avoiding bulky metallic fixtures and rigid sample geometries, while they still provide high-resolution dielectric measurements. Meanwhile, Planar microwave sensors probe materials at relevant GHz frequencies, where polarization mechanisms, ionic effects, and dispersion behaviors are more pronounced, and where contactless or near-contact sensing is achieved with reduced electrode polarization. These advantages position planar microwave sensors as a useful and mature solution for nondestructive dielectric measurements across solids, liquids, and microfluidic samples.

**Figure 1:**
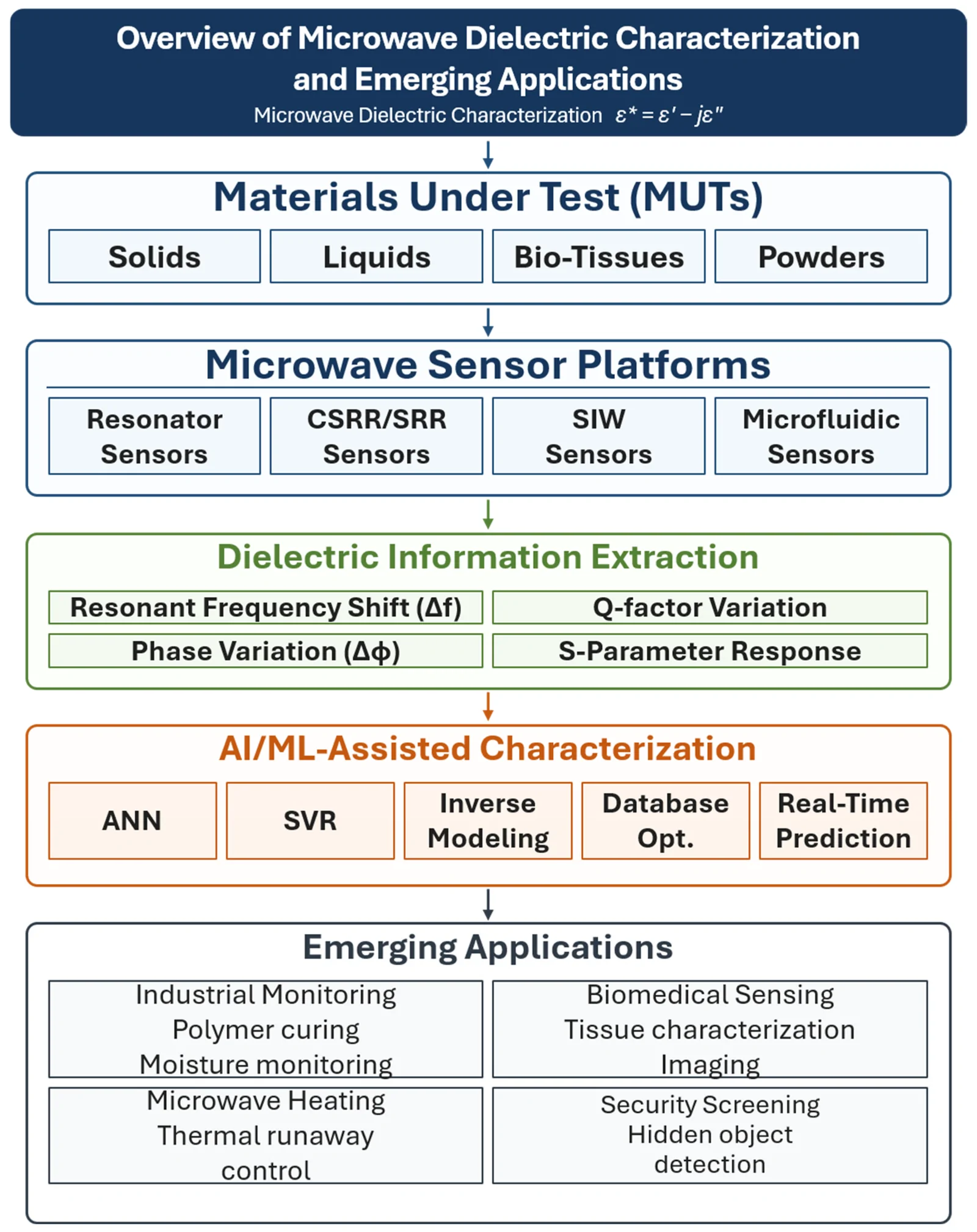
Overview of microwave dielectric characterization and its emerging applications.

Recent literature on planar microwave dielectric sensing reveals three broad shifts driven by both scientific and engineering requirements. First, many systems are moving from broadband characterization toward single-frequency or narrowband optimization, which simplifies readout and enables faster monitoring. Second, field-confining resonant and interferometric structures are being used to amplify small dielectric perturbations. Third, differential and phase-variation schemes are increasingly adopted to reject common-mode drift [45,46,47,48,49,50,51]. These trends must, however, be evaluated against the associated calibration burden, fabrication sensitivity, environmental stability, and manufacturing cost; sensitivity or Q-factor alone does not establish practical superiority. Figure 1 therefore presents the review as a design chain from material and sample interface to sensor architecture, dielectric-information extraction, data processing, and application.

Representative contributions from the research groups highlighted in the recent literature show complementary routes toward practical dielectric characterization. The Katia Grenier group, often in collaboration with Ferran Martín, has advanced highly sensitive open complementary split ring resonators (OCSRRP) and differential microfluidic sensors for liquid dielectric characterization and biomolecular monitoring [52,53]. The Mohammad Zarifi group has developed ultra-high-resolution, active-resonator, and feedback-assisted planar sensing strategies, including data-driven resolution enhancement for low-concentration liquids [54,55,56]. The Ferran Martín group has systematically developed frequency- and phase-variation, DGS, and single-frequency sensing methods for quantitative permittivity extraction [45,52,53,57,58,59]. The Kamran Ghorbani group has contributed differential, phase-, amplitude-, and coupler-based architectures for sensitive characterization of solid and liquid dielectrics [45,59,60,61,62]. The Carlos G. Juan group has demonstrated planar open-loop and coupled-resonator sensors for permittivity measurement and simultaneous multi-parameter extraction [63,64]. Together, these studies illustrate the evolution from isolated resonator demonstrations toward robust single-frequency, differential, active, and microfluidic measurement platforms.

Microwave sensors extract dielectric properties (ε′ and ε″) from the interaction between electromagnetic fields and materials under test (MUTs). Various sensing platforms, including resonator-, metamaterial-, substrate-integrated-waveguide (SIW)-, and microfluidic-based sensors, convert dielectric perturbations into measurable responses such as resonant-frequency shift, phase variation, Q-factor change, and S-parameter variation. Recent advances further integrate machine learning and inversion algorithms for real-time characterization and intelligent prediction, enabling applications in industrial monitoring, biomedical sensing, microwave-assisted processing, and security screening.

### 1.2. Scope, Positioning, and Contributions

In this review, planar microwave sensors are defined as permittivity-sensing structures implemented in a two-dimensional printed format, typically using microstrip or coplanar-waveguide technologies and standard PCB processes. The core taxonomy includes transmission-line resonators, ring and stub resonators, interdigital-capacitor structures, SRR/CSRR and DGS elements, SIW/SISL platforms, interferometric configurations, and their microfluidic interfaces. The primary focus is the nondestructive extraction of dielectric constant, loss tangent, or effective conductivity for solid, liquid, and microfluidic samples.

To preserve this focus, microwave imaging, RFID, radar, waveguide, and tomography methods are treated as boundary or system-level comparators rather than as classes of planar sensors. They are included only where they contribute transferable concepts—such as spatial sampling, inverse reconstruction, temperature-tolerant measurement, or low-cost readout—that directly inform the design and deployment of planar dielectric sensors. This difference is made explicitly in Section 3.4.

Several recent reviews have summarized microwave permittivity sensors, planar resonator topologies, extraction techniques, applications, and open challenges [24,65,66]. The added value of the present review is a design-oriented synthesis that connects five levels of decision making: (1) physical architecture, (2) operating principle, (3) readout and extraction mechanism, (4) sample interface, and (5) target application. Beyond cataloging reported performance, the review evaluates why an approach is preferable under a given sensing scenario and identifies the compromises in sensitivity, dynamic range, repeatability, fabrication yield, calibration complexity, environmental robustness, cost, and scalability.

Accordingly, Section 2 and Section 3 progress from physical mechanisms and performance metrics to a hierarchical architecture/readout/sample classification, while the new Section 4 consolidates engineering trade-offs, reproducibility practices, PCB tolerance effects, aging, and industrial-deployment requirements. This organization is intended to provide both a critical comparison of the literature and an actionable selection framework for researchers and measurement-system designers.

## 2. Fundamentals of Dielectric Measurements Using Planar Microwave Sensors

### 2.1. Dielectric Properties at Microwave Frequencies

At microwave frequencies, the dielectric response of a material is described by complex permittivity ε* = ε′ − jε″, where the real part ε′ represents the material’s ability to store electric energy through polarization, while the imaginary part ε″ quantifies dielectric loss associated with energy dissipation. A convenient measure of loss is the loss tangent, tanδ = ε″/ε′, which determines the attenuation of electromagnetic waves inside the material. In planar microwave resonant sensors, these two components show distinct measurable performance. Changes in ε′ primarily alter the effective capacitance and stored electric energy, leading to a shift in resonant frequency. Increases in ε″ result in additional loss that broadens the resonance bandwidth and reduces the quality factor (Q-factor) and reduces amplitude contrast and sensitivity. Both ε′ and ε″ can be extracted from resonance behavior or broadband S-parameter response [67,68,69,70]. However, many industrial monitoring tasks primarily require accurate measurement of ε′ at a single operating frequency, since ε′ strongly correlates with composition, concentration, or moisture content. Consequently, modern planar sensor designs place greater emphasis on high-resolution, single-frequency measurement of ε′. Loss estimation is secondary when applications involve high-loss materials [45,71,72].

**Figure 2:**
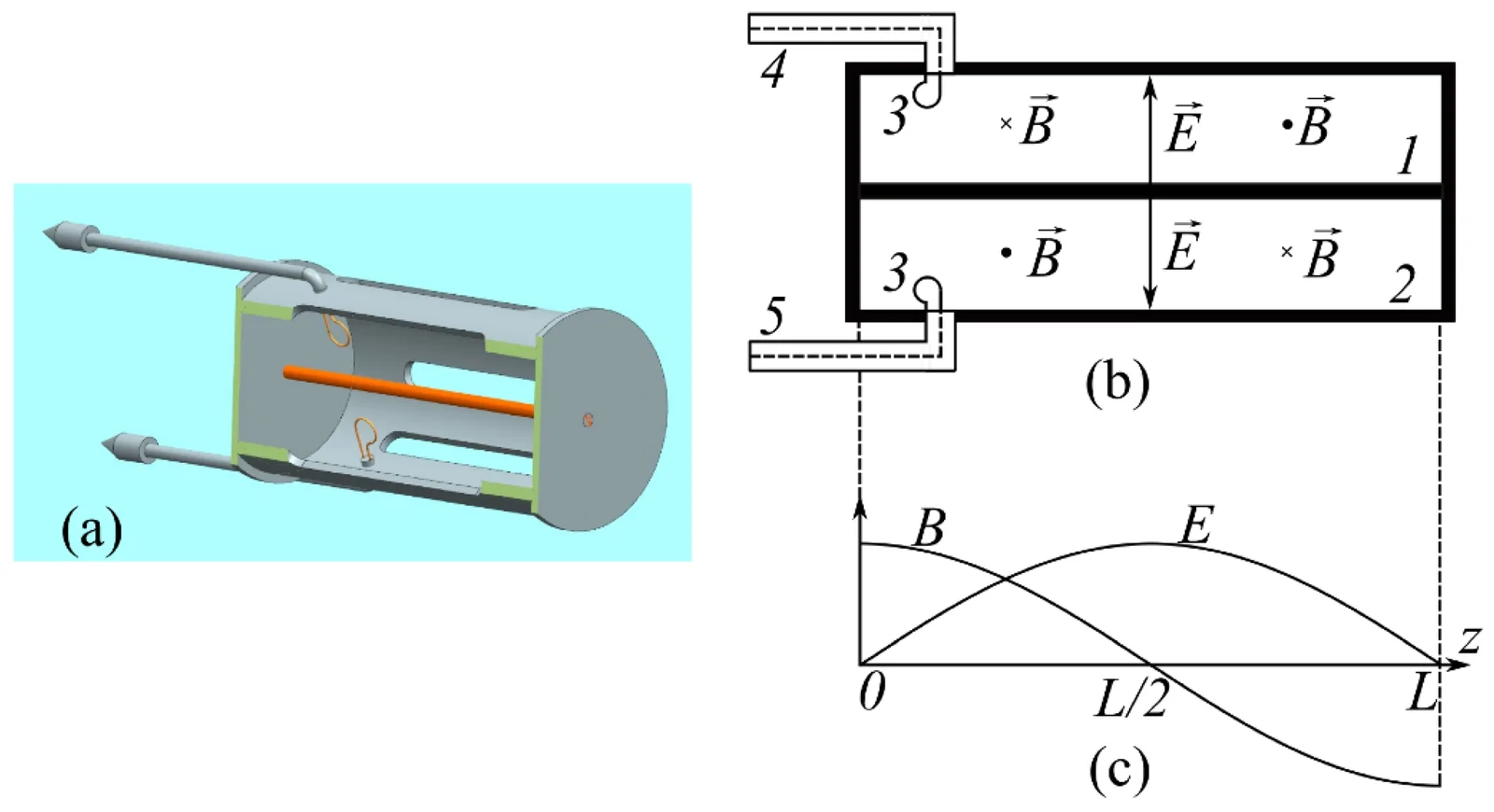
Typical resonant microwave cavity sensor and field distribution for permittivity-sensitive measurement, adapted from Galka et al. [74]. (**a**) 3D model; (**b**) cavity diagram; (**c**) distribution of electric E and magnetic B fields.

Dielectric properties are usually frequency-dependent since the dominant polarization and loss mechanisms respond differently across the electromagnetic spectrum, leading to dispersion in ε′ and ε″. Broadband dielectric spectroscopy is valuable in measuring permittivity over a wide frequency range, and can reveal relaxation dynamics, distinguish material phases, and enable multi-parameter identification of composition. This is useful for complex mixtures, biological media, and heterogeneous composites. However, for practical sensing and process monitoring, broadband approaches often have substantial drawbacks, including increased instrumentation complexity, larger data volumes, greater sensitivity to systematic errors, and the need for more accurate calibration. In many industrial and biomedical applications, the primary objective is not to reconstruct the full dispersion curve but to track a material state variable, such as moisture content, concentration, or degree of cure. It correlates strongly with permittivity at a single chosen frequency [73,74,75]. In resonant microwave sensors, variations in the dielectric properties of the material under test perturb the stored electromagnetic energy and lead to measurable changes in resonant frequency, bandwidth, phase response, and Q-factor. A representative cavity-based configuration and its field distribution are illustrated in Figure 2 [74]. Higher-order modes in balanced-type circular-disk resonator structures facilitate high-precision permittivity sensing from 110 to 170 GHz [76]. Single-frequency or narrowband sensing thus offers clear advantages. It enables faster measurement and real-time feedback, simplifies calibration and data interpretation, improves robustness, and supports compact low-cost electronics. They can replace laboratory vector network analyzers in some situations. This practical inclination has shaped the evolution of planar microwave dielectric sensors toward designs that maximize sensitivity and stability at a specific operating point. The core measurement principle is the near-field perturbation of a resonant or interferometric structure by the material under test.

### 2.2. Interaction Mechanisms and Perturbation

Planar microwave dielectric sensors primarily operate via near-field interactions [46,77]. The electromagnetic fields are strongly localized in specific regions of a planar transmission line or resonant structure, such as coupling gaps, interdigital capacitors, split-ring apertures, or defected ground slots, and vanish rapidly away from these metal traces. When a material under test (MUT) is brought into these regions, its permittivity affects the local electric-field distribution and changes the effective capacitance and inductance experienced by the sensor. This perturbation changes the electromagnetic energy storage and dissipation in the structure, leading to measurable variations in its scattering parameters. In resonant sensors, the most common indicator is a shift in resonant frequency, primarily due to changes in ε′. The changes in resonance depth and bandwidth reflect increased loss (ε″ and conductivity) and reduced Q-factor. In non-resonant configurations, the MUT-induced perturbation can appear as changes in transmission magnitude or as highly sensitive phase variations when the sensor is designed to operate near the steep phase slope. The interaction is localized, and near-field sensing enables nondestructive and often contactless measurements with small sample volumes. These principles motivate the widespread use of resonant gaps, metamaterial-inspired elements, and differential or phase-based readout schemes in modern planar microwave sensors. They obviously maximize the overlap between the near fields and the MUT to enhance dielectric sensitivity [47,78]. Representative OCSRR, DGS, slow-wave, and branch-line-coupler implementations further demonstrate how field localization and dispersive phase response can be deliberately engineered to increase dielectric sensitivity [45,52,57,58,62].

The sensor response can be understood using perturbation theory, which relates resonance changes to the difference between the electromagnetic energy stored in the unperturbed structure and that stored after dielectric loading. Qualitatively, a higher ε′ of MUT increases the effective capacitance and strengthens electric-field energy storage in the sensing region, thereby shifting the resonant frequency. The increased dielectric loss (higher ε″ or conductivity) introduces additional dissipation and lowers the Q-factor. The magnitude of this frequency or phase perturbation is strongly influenced by three coupled factors: (a) the permittivity contrast between the MUT and the surrounding medium, (b) the spatial overlap between the MUT and the sensor electric/magnetic energy density, and (c) the effective volume of the MUT within the near-field penetration depth. Consequently, field confinement becomes the central design lever for achieving high sensitivity. It is important to concentrate the electric field into subwavelength gaps or resonant apertures; using metamaterial-inspired structures, defected ground patterns, or capacitive loading, one can maximize the energy fraction interacting with the MUT and amplify small dielectric variations into resolvable shifts in frequency, amplitude, or phase [46,79,80].

### 2.3. Performance Metrics

Sensitivity and resolution are the primary performance descriptors of planar microwave dielectric sensors. It is typically quantified in either frequency- or phase-based forms, depending on the readout mode. For resonant sensors, the frequency sensitivity is commonly expressed as Sf = Δf/Δε′, whereas for interferometric or phase-based sensors, the phase sensitivity is defined as Sϕ = Δϕ/Δε′, often evaluated at a fixed frequency. The phase slope is usually maximized. These sensitivities directly determine the smallest resolvable change in dielectric constant. It also depends on the measurement noise floor, instrument resolution, and environmental drift [48,68,81]. In addition to sensitivity, the resolution and dynamic range of a sensor are crucial. Resolution indicates the minimum detectable change in permittivity. It is often limited by phase noise or frequency tracking uncertainty. The dynamic range specifies the maximum permittivity span over which the extraction model remains accurate and monotonic. Achieving high sensitivity generally requires a high quality factor and strong electromagnetic field confinement in the sensing region. Hybrid configurations with coupled SRR-ELC elements shielded by metasurfaces have demonstrated a high Q-factor (up to 171) at 3.36 GHz, reaching high sensitivity for characterizing solid materials [82]. However, these same design choices can amplify susceptibility to practical non-idealities, such as temperature drift, sample-positioning errors, and fabrication tolerances.

End-users evaluate planar microwave dielectric sensors using a broader set of measurement systems that reflect deployment conditions. Reported measurement error and reproducibility across repeated trials determine whether a sensor is suitable for quality control or biomedical tests. The required sample volume and sample-handling constraints are also important, particularly for microfluidic and biomedical applications where only microliter or nanoliter volumes may be available [83,84,85]. Furthermore, the calibration and extraction method strongly impacts robustness, ease of implementation, and sensitivity to systematic bias. It relies on simple linear regression, simulation-assisted inversion, or differential- or phase-based processing. Environmental robustness is equally critical. Temperature stability can be a limiting factor because both the substrate permittivity and the sample properties depend on temperature. Fabrication tolerance is especially important for high-confinement geometries with narrow gaps. Small dimensional errors can shift the operating frequency and distort calibration curves. These considerations motivate the comparative tables presented later in this review. Sensors are benchmarked not only by sensitivity and operating frequency but also by accuracy, repeatability, sample compatibility, and ease of fabrication [45,46,67,77,86,87].

### 2.4. Measurement Setup and Common Practice

In experimental studies of planar microwave dielectric sensors, a vector network analyzer (VNA) commonly measures S_11_ or S_21_ over a band surrounding the operating point. The observed changes in frequency, amplitude, phase, bandwidth, and Q-factor are influenced by the complete measurement chain rather than by the sensor alone. Major error sources include VNA drift and calibration residuals; connector repeatability; cable bending and temperature; launch, adapter, and fixture parasitics; grounding and contact-pressure variation; sample-position, volume, thickness, and air-gap errors; bubbles in microfluidic channels; and temperature or humidity dependence of both the MUT and the substrate [87,88]. These effects become important for high-Q or strongly confined structures since small perturbations can be comparable to the measure-induced response.

A protocol should first define the calibration plane and use a method appropriate to the fixture, such as SOLT, TRL, or a fixture-specific de-embedding procedure. Connectors should be mated with controlled torque; cables should be strain-relieved or replaced by semi-rigid interconnects; sample holders should provide mechanical registration and controlled contact pressure; and liquid channels should be inspected for bubbles and volume variation. Blank, reference, or differential channels can suppress common-mode drift, while stable reference materials can verify the measurement chain before and after a test sequence [45,46,47,48,49,50,51,88].

For quantitative claims, studies should report replicate measurements, within-device repeatability, between-device and between-batch reproducibility, environmental conditions, calibration interval, and an uncertainty budget that separates instrument, fixture, sample-handling, model, and fabrication contributions. Independent validation with reference materials or a second characterization method is preferable to reporting only a calibration-curve fit.

## 3. Hierarchical Classification and Critical Comparison of Planar Microwave Sensors

The classification used in this review is hierarchical rather than a mixture of structures, algorithms, and applications. Level 1 specifies the physical architecture (transmission-line, resonant, metamaterial-inspired, DGS, SIW/SISL, interferometric, or hybrid). Level 2 identifies the operating principle (resonant perturbation, broadband propagation, coupling, or interference). Level 3 specifies the readout (frequency shift, Q-factor or amplitude change, phase variation, differential response, or multi-frequency fitting). Level 4 describes the sample interface (solid contact, contactless standoff, bulk liquid, or microfluidic channel), and Level 5 maps the resulting system to representative applications. AI/ML is treated as a cross-cutting inversion and decision layer, whereas imaging and radar are treated as system-level boundary examples.

### 3.1. Architecture and Operating Principle Taxonomy

At the highest level, planar microwave dielectric sensors are resonant or non-resonant, but this binary distinction must be connected to the sensing task. Resonant microstrip, CPW, SRR/CSRR, DGS, SISL, and SIW structures provide sharp frequency or phase features and are well suited to detecting small changes over a limited permittivity range. Their advantages are compactness, straightforward single-frequency tracking, and strong field localization; their principal compromises are narrow bandwidth, sensitivity to sample placement and fabrication error, and a greater need for temperature and drift control. Non-resonant transmission-line sensors provide broadband amplitude and phase information that can capture dispersion and support simultaneous extraction of multiple dielectric parameters, but usually require more accurate de-embedding, a larger dataset, and a more complex inversion model [67,89,90,91].

**Figure 3:**
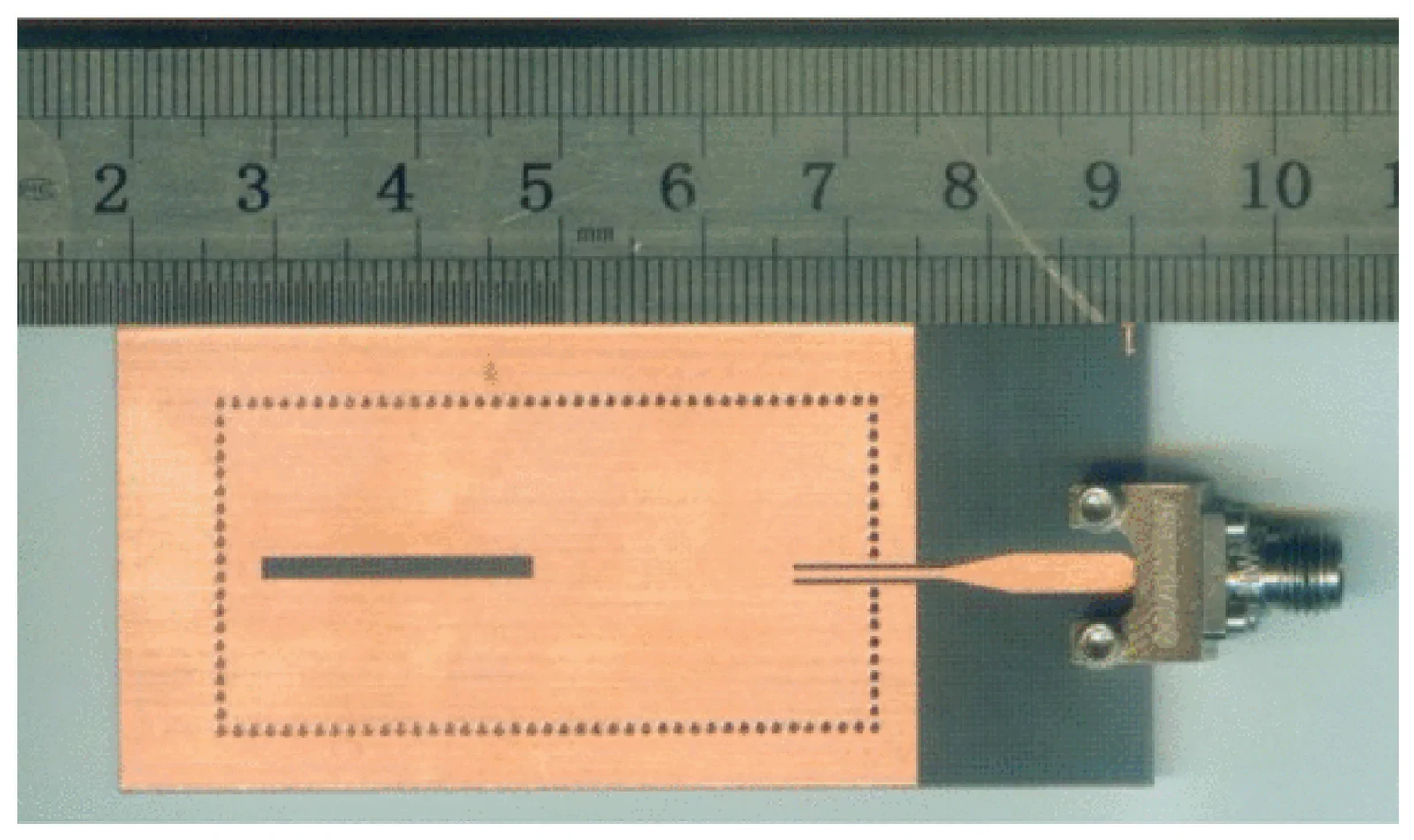
An SIW resonator sensor for liquid permittivity measurements at C band [92].

Within the resonant class, the preferred architecture depends on the dominant constraint. Microstrip and CPW resonators are inexpensive and easy to integrate but expose the field to the environment. SRR/CSRR and DGS structures intensify the local electric field and reduce sample volume, yet narrow gaps and etched slots increase tolerance sensitivity. SIW and SISL platforms provide higher Q-factor, shielding, and lower radiation loss, but require via fences, multilayer alignment, or more specialized fabrication. Figure 3 illustrates a C-band SIW resonator sensor, in which a slot is introduced to enable the measurement of liquid permittivity. Interferometric, phase-based, and differential configurations can increase fixed-frequency sensitivity and reject common-mode drift, although their performance depends on phase noise, channel matching, and operating-point control [45,46,47,48,49,50,51]. Thus, no architecture is universally superior; the appropriate choice is determined by whether the priority is absolute permittivity accuracy, small-change detection, broadband dispersion, minimal sample volume, low-cost manufacture, or field robustness. Recent single-frequency studies also show that sensitivity optimization must be matched to the selected frequency-, phase-, amplitude-, differential-, or coupled-resonator readout rather than treated as a topology-independent quantity [45,57,58,59,60,61,62,63,64].

Table 1 should therefore be interpreted as a design comparison rather than a ranking by sensitivity. High-Q SIW/SISL sensors are attractive when stable fixtures and controlled fabrication are available; compact CSRR/DGS sensors are effective for small samples and relative or threshold measurements; differential and reflective phase-variation systems are preferable when environmental drift or low-cost fixed-frequency readout is a dominant concern; and broadband transmission methods remain valuable when dispersion or simultaneous extraction of ε′ and ε″ is required. Reported errors below approximately 3–5% are promising, but direct comparison is limited by differences in calibration materials, permittivity range, sample geometry, replicate count, temperature control, and whether validation was performed on the same device used for model fitting.

### 3.2. Sample Interfaces: Microfluidic-Integrated Planar Sensors

Microwave planar sensors, such as split-ring resonators (SRRs), complementary split-ring resonators (CSRRs) [105,106], and substrate-integrated waveguides (SIWs), are important for high-precision dielectric characterization [107] from biomedical monitoring to industrial quality control [46,65,67,101]. These sensors rely on the perturbation principle, where placing an MUT in regions of high electric-field concentration induces shifts in resonance frequency and Q-factor [67,101,110]. Innovations such as modified two-turn rectangular spiral resonators (M2TR-SR) and extended horizontal microstrip lines (EH-ML) further enhance sensitivity. They create stronger fringing fields and improve resolution through low capacitive coupling. Furthermore, to reduce cross-sensitivity from environmental factors, such as temperature, differential measurement processes are integrated into unit structures [50,51,108]. Unwanted frequency shifts are removed, thereby enhancing measurement accuracy. These advancements enable a wide range of applications, including non-invasive blood glucose sensing from fingertips [111,112,113], electrolyte concentration monitoring [114], the detection of metals in polluted water, and the real-time analysis of liquid chemical concentrations in microfluidic channels. Furthermore, interconnected SRRs integrated with disposable 3D-printed sample cups and machine learning algorithms have demonstrated high sensitivity in characterizing binary ethanol–water solutions [115]. Recent examples include a fully 3D-printed SIW-based glucose sensor with a compact sample holder and microfluidic-compatible readout [116], as well as multichannel microwave readout electronics for real-time microplastics assessment in aqueous environments [117].

IDC-enhanced microwave planar sensors improve detection sensitivity by integrating interdigital capacitors (IDCs) into resonant structures, such as SRR or electric-LC (ELC) designs. Furthermore, the 3D metallic walls around integrated IDCs have been shown to increase the concentration of electric fields in space, thereby amplifying the frequency shift compared to traditional SRR configurations [118]. Additional capacitive loading is introduced through the interdigital fingers of the IDC. These sensors can gather and concentrate much stronger electric fields into highly localized “hot spots” near the gaps. This intense field confinement is governed by the perturbation principle. The sample must be placed in the sensing area of maximum field strength. Then, it induces shifts in the resonance frequency and quality factor. This localization is advantageous for the characterization of thin layers and small-volume liquid samples (as little as 2.5 µL). Furthermore, these IDC-enhanced structures enable highly accurate dielectric characterization across diverse applications, ranging from monitoring glucose levels in aqueous solutions to high-precision analysis of solid materials and food quality. For anisotropic materials such as liquid crystals, implementing an electric field direction is crucial; recent works have demonstrated that epsilon-near-zero material tunnels provide the necessary field alignment to accurately distinguish between parallel and perpendicular dielectric properties [109]. IDC-DGS implementations can also be functionalized with hydrophilic polymer composites, enabling high-sensitivity humidity sensing via changes in resonance frequency and transmission magnitude [119].

The studies summarized in Table 2 show that microfluidic integration is most advantageous when sample scarcity, contamination control, and repeated handling are central constraints. High-Q resonators can improve resolution for microliter samples, whereas differential or interferometric channels better suppress temperature and fixture drift [50,80,111]. These gains carry application-specific penalties: channel height and position become part of the electromagnetic calibration; bubbles, residue, evaporation, flow-rate variation, and polymer swelling can dominate repeatability; and disposable channels improve hygiene but introduce device-to-device dimensional variation. Consequently, microfluidic sensors should be compared using not only sensitivity and sample volume, but also filling reproducibility, cleaning or disposability, temperature control, and between-channel variability. Differential microfluidic and active-resonator implementations reported by the Grenier, Martín, Zarifi, and Ghorbani groups illustrate this balance between field overlap, sample handling, resolution, and robustness [52,53,55,59,61].

### 3.3. Data and Inversion Layer: AI/ML-Assisted Characterization

AI and machine learning are best regarded as a cross-cutting data and inversion layer rather than as a sensor architecture. They can approximate nonlinear mappings between measured S-parameters and dielectric properties, combine multiple features for classification, and compensate for coupled variables that are difficult to treat analytically [33,122,123]. Their most compelling use cases include amplitude-only or low-cost readout, multi-parameter inversion, rapid process monitoring, and classification when the training domain is clearly defined. Data-assisted resolution enhancement is not new to this field; fuzzy-neural processing has also been used to improve the robustness and effective resolution of planar resonator measurements [54].

**Figure 4:**
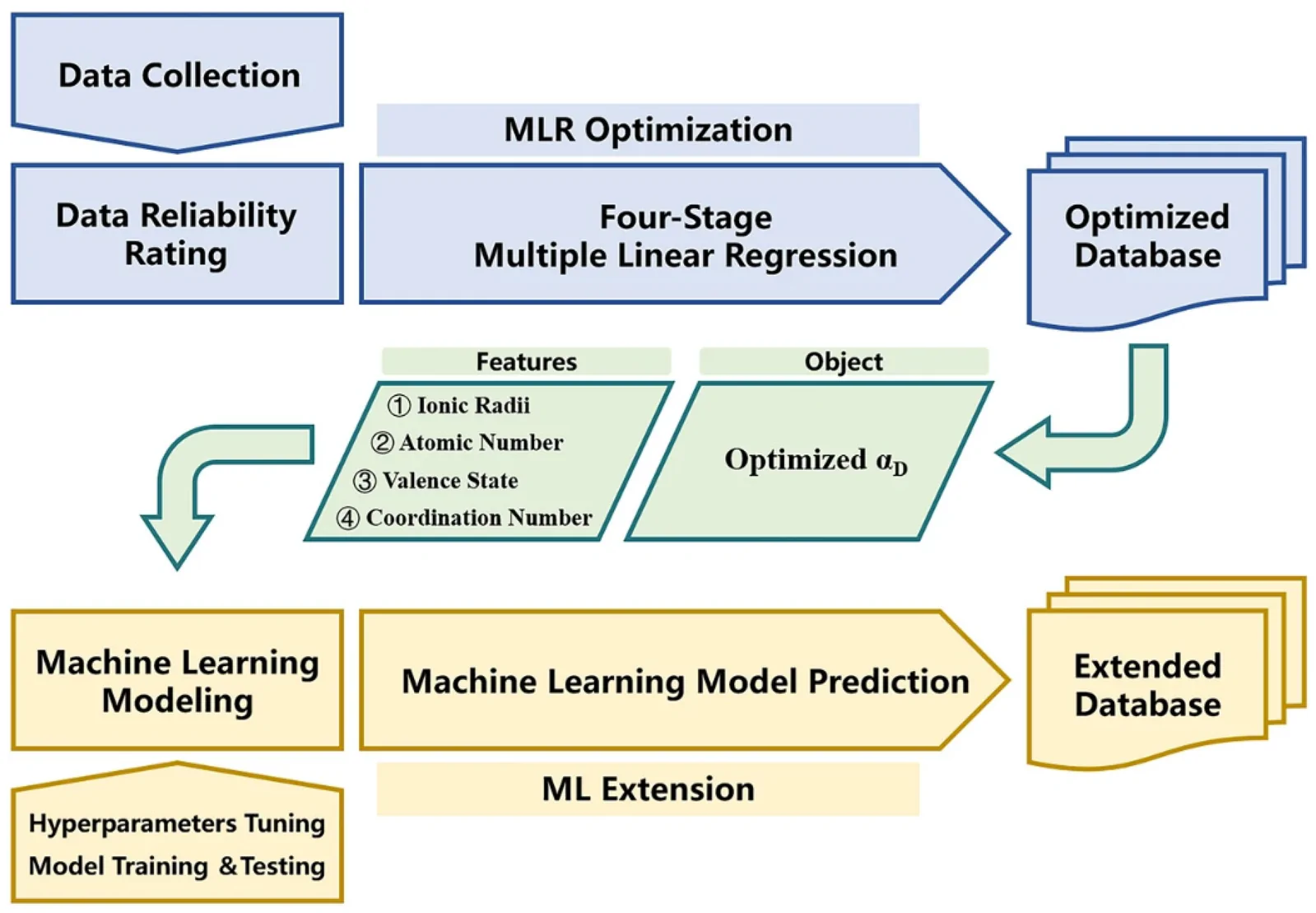
Procedures of the multiple linear regression optimization and ML extension of the ion dielectric polarizability database [34].

In industrial and real-time monitoring applications, AI-driven technologies provide robust solutions for process control and safety. An integrated system based on an oblique aperture ridge waveguide and ANNs enables the continuous monitoring of complex permittivity during microwave heating, which is essential for preventing thermal instability and runaway [29]. Similarly, for real-time evaluation in tunnel construction, GA optimized UWB impulse radar systems can extract the electrical and physical parameters of buried objects nearly 29 times faster than traditional mean squared error methods [104]. Furthermore, machine learning has been utilized to optimize dielectric polarizability databases, enabling the prediction of the relative permittivity of various microwave ceramics to support the next-generation 5G network components [34], as shown in Figure 4.

The biomedical field has also seen substantial progress through the application of supervised learning and deep neural networks. Multi-layer perceptron (MLP) neural networks have been successfully applied to quantitative microwave breast imaging [11]. By combining MLP-NN with skin surface rejection preprocessing, 4D scattered data can be converted into 3D complex permittivity profiles for accurate tissue characterization. Moreover, various supervised learning algorithms, such as support vector machines (SVMs), random forest, and k-nearest neighbors, have demonstrated high accuracy in vivo tissues. Research indicates that SVM algorithms can differentiate between malignant, benign, and normal mammary tissues with a median accuracy of 94.4%, highlighting the potential of microwave dielectric measurement as a diagnostic tool [12].

These benefits do not remove the underlying measurement constraints. Most reported models are application-specific and depend on training data that adequately span material composition, permittivity and loss ranges, sensor-to-sensor fabrication variation, sample placement, temperature, humidity, and instrument drift. Simulation-generated data can reduce experimental cost but may create a simulation-to-measurement domain gap. Models trained on one sensor batch, fixture, or material family can fail when the hardware or operating environment changes. In quality-critical applications, limited interpretability and the absence of calibrated prediction uncertainty can make a numerically accurate model difficult to audit.

Edge deployment introduces additional constraints on memory, latency, power consumption, and model maintenance. Practical studies should therefore report dataset composition and size, the separation of training/validation/test sets, independent validation across hardware batches and environmental conditions, prediction uncertainty, computational cost, and recalibration or transfer learning procedures. Physics-informed features, uncertainty-aware models, compact networks, and hybrid analytical–ML inversion are promising because they can reduce data demand and improve traceability, but they should be benchmarked against transparent regression or physics-based baselines.

### 3.4. Boundary of Scope: Imaging and System-Level Nondestructive Evaluation

**Figure 5:**
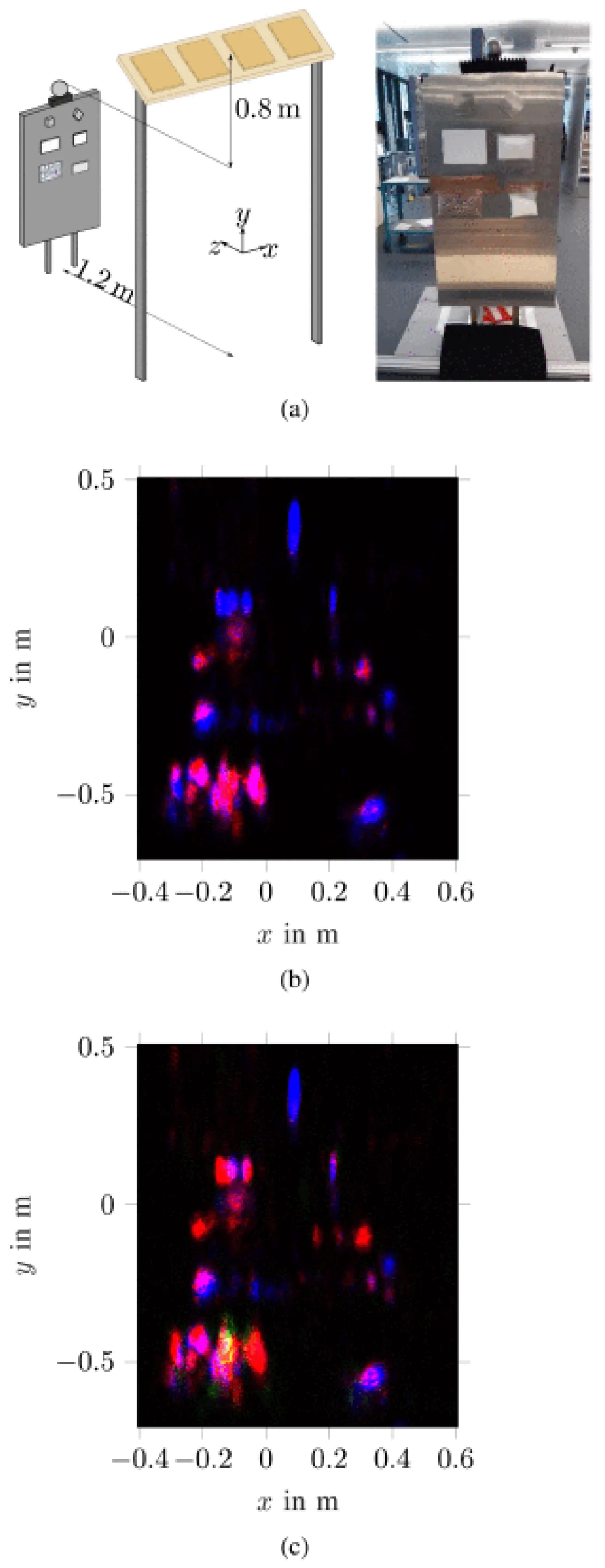
(**a**) Test setup and image from test structures placed onto a metal plate; (**b**) conventional results; (**c**) the close-range adapted decomposition technique [13].

Microwave imaging, RFID, radar, and tomography are not classified here as planar dielectric sensors because they use spatially distributed measurements and system-level inverse reconstruction. They are retained in this section only to identify transferable concepts relevant to planar platforms: contactless examination, multipath-robust feature extraction, rapid inverse modeling, and the extension from local measurements to spatially resolved material assessment. This bounded treatment prevents these technologies from weakening the central architecture-based classification. A representative example is the polarimetric microwave imaging approach reported by Root et al. [13] for walk-through security scanning. As shown in Figure 5, the close-range adapted target-decomposition method provides improved discrimination of the measured scattering responses compared with the conventional polarimetric decomposition approach.

**Figure 6:**
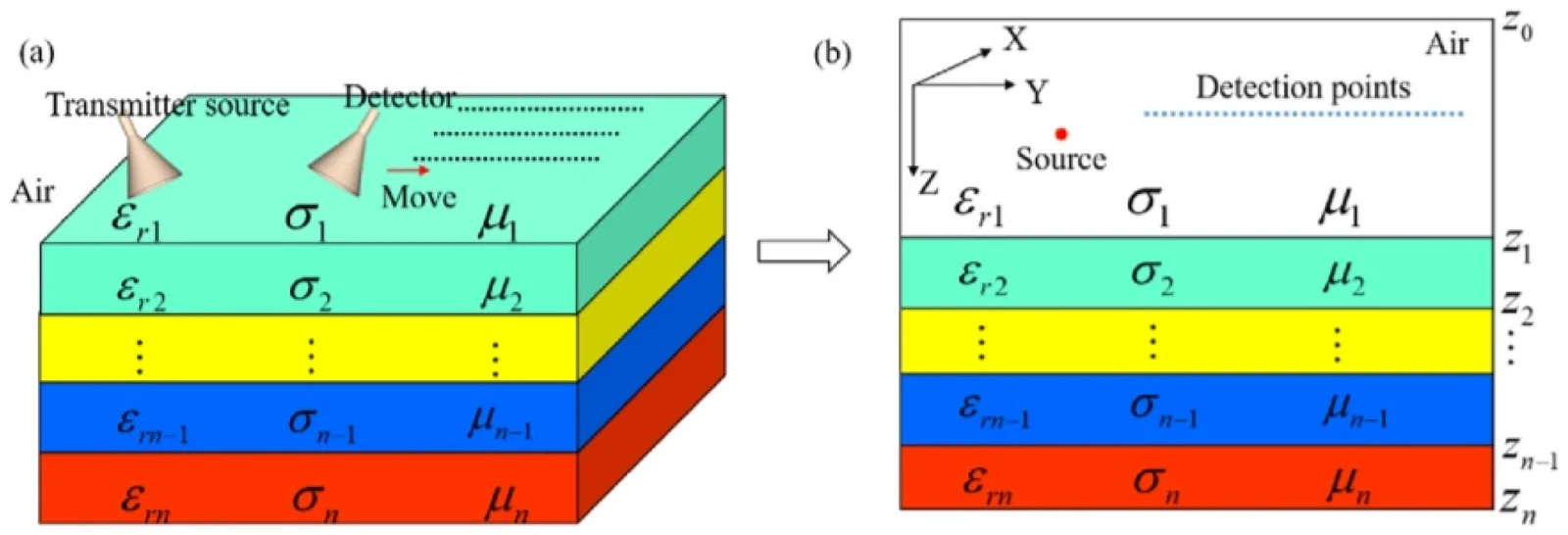
A typical microwave detection system for stratified media [31]. (**a**) schematic of the practical scanning configuration; (**b**) corresponding theoretical layered-medium model.

Microwave NDE also provides robust solutions for the industrial measurement of composite materials. A multi-parameter inversion method based on the dyadic Green’s function allows for the simultaneous reconstruction of relative permittivity and conductivity profiles in layered media [31], as shown in Figure 6. Utilizing the Distorted Born Iterative Method (DBIM) can identify minor internal imperfections and subsurface attachments, which is critical for ensuring the structural integrity of components in aviation, ship building, and bridge construction. Furthermore, advanced algorithms such as DIMAR (Contactless Material Identification Algorithm) have been introduced for the fine-powdered identification of dielectrics via moving UHF RFID systems [32]. By theoretically analyzing the influence of material reflection on received signal strength (RSS) from an electric field perspective, DIMAR provides a high-resolution identification framework that remains robust in multipath environments using evolutionary optimization strategies.

In the field of heavy industrial operations, real-time characterization of the tunnel face is essential for the safety of tunnel boring machine processes. Ultra-wideband (UWB) impulse radar technology along with genetic algorithm (GA) optimization enables the near-instantaneous extraction of complex permittivity for buried objects and geological features. These systems can identify buried oil or sewer pipes and characterize the surrounding soil conditions at processing speeds nearly 29 times faster than traditional mean squared error methods [104]. Additionally, microwave sensors have demonstrated unique capabilities in biological NDE, such as the real-time monitoring of subsurface bacterial growth using passive biosensors that detect changes in the dielectric properties of the growth medium [110]. These advancements demonstrate that advanced imaging algorithms and high-sensitivity microwave architectures expand the applications of planar sensors to real-world monitoring.

These techniques are used as examples and not as a performance comparison. Imaging, RFID, and radar contribute inversion and spatial-sampling strategies, MEMS and optical-waveguide platforms offer package-level concepts for thermal reference, miniaturization, and multimodal integration [124,125,126], and high-frequency communication materials motivate accurate permittivity data [127]. The performance tables and design recommendations in this review remain centered on microwave planar sensors. Recent examples clarify this boundary: square split-ring resonators can directly detect nanoparticle concentrations in PVDF-based nanocomposites [128], whereas ultra-wideband water-immersion antennas used for underwater ultrasonic sensing in microwave-induced thermoacoustic tomography represent a system-level sensing platform [129].

## 4. Engineering Trade-Offs, Reproducibility, and Industrial Deployment

### 4.1. Architecture Selection and Trade-Off Synthesis

It is known that increasing Q-factor, field confinement, or phase slope improves nominal sensitivity of sensors. However, it also narrows the usable range, amplifies dimensional and temperature perturbations, increases settling and tracking requirements, and reduces fabrication yield. Table 3 summarizes the conditions under which the principal architecture/readout combinations are preferable and the controls required to realize their reported performance.

Table 3 also clarifies why reported sensitivity values should not be compared without context. A lower-sensitivity differential sensor may provide a better field detection limit than a high-Q single resonator if the latter is dominated by temperature or connector drift. Conversely, an SIW or high-Q resonator can provide superior absolute characterization when the calibration, fixture, and manufacturing process are controlled. The relevant figure of merit is therefore the uncertainty-limited resolution over the required dynamic range and operating environment, not sensitivity alone.

### 4.2. Calibration, Uncertainty, and Reproducibility

Calibration should be treated as a traceable measurement process rather than as a single curve-fitting step. The calibration plane, standards, fixture de-embedding, reference temperature, and sample-handling protocol must be consistent with the intended deployment. Table 4 links common error sources to their measurement signatures, practical mitigation, and minimum reporting information.

At minimum, a validation dataset should include repeated loading of the same MUT, independently prepared samples, multiple sensor devices, and—where industrial use is claimed—more than one fabrication batch. The reported uncertainty should be associated with the complete extraction result, not only the VNA specification. A useful distinction is repeatability (same device, operator, and conditions), intermediate precision (different days, operators, or instruments), and reproducibility (different devices, batches, or laboratories).

### 4.3. PCB Fabrication Tolerances and Scalable Manufacturing

Planar sensors convert small geometric and material variations into electromagnetic shifts, so the same field confinement that improves sensitivity can reduce manufacturing tolerance. Under- or over-etching changes gap widths, ring dimensions, and DGS slots; copper thickness and surface roughness modify conductor loss; substrate thickness, permittivity, and loss tangent vary between datasheet values, production lots, and local regions; and via diameter, pitch, plating, multilayer registration, solder volume, and connector launches affect SIW and high-Q structures. These variations alter the unloaded resonance, Q-factor, field distribution, and ultimately the calibration function and device yield.

For low-cost FR-4, nominal substrate parameters are often adequate for qualitative, relative, or threshold sensing, especially when differential/reference channels are used. They are less reliable for high-accuracy absolute permittivity extraction unless the actual board material and fabricated geometry are characterized. Practical measures include designing gaps and vias with process margin; adding resonator or transmission-line coupons to each panel; measuring critical dimensions; characterizing each substrate lot; using per-batch or per-device calibration when necessary; and performing tolerance or Monte Carlo analysis during design. Scalability should be reported in terms of yield, between-device spread, calibration transferability, and the amount of device-specific trimming or calibration required—not only by the nominal PCB cost.

### 4.4. Sensor Aging, Long-Term Drift, and Recalibration

Long-term deployment introduces mechanisms that are rarely visible in short laboratory demonstrations. Moisture uptake and thermal history can change substrate properties; copper oxidation, solder and connector relaxation, adhesive creep, protective-coating changes, channel swelling, residue, repeated cleaning, and biofouling can modify loss, coupling, or sample position. Reference and sensing channels may also age at different rates, so an initially balanced differential design is not automatically drift-free. These effects can produce slow baseline motion, reduced Q-factor, altered sensitivity, and calibration-curve drift.

Industrial validation should include thermal and humidity cycling, storage and operating-time studies, repeated connection or cleaning cycles, contamination tests appropriate to the application, and periodic measurement of stable reference materials. Useful lifecycle metrics include baseline drift per unit time or cycle, sensitivity drift, time to recalibration, failure or replacement criteria, and recovery after environmental stress. Replaceable sensing coupons, sealed reference resonators, embedded temperature/humidity monitoring, self-test standards, and scheduled or condition-based recalibration can reduce maintenance burden. Long-term stability must be treated as a design parameter and reported together with sensitivity and accuracy.

### 4.5. Translation from Prototype to Measurement System

A practical development pathway is to define the measured quantity and environment first; select an architecture using uncertainty-limited resolution rather than nominal sensitivity; establish a calibration and sample-handling protocol; design for fabrication margin and batch verification; validate repeatability, reproducibility, and lifecycle drift; and only then add AI/ML where it provides measurable benefit over transparent analytical or regression baselines. This sequence prevents computational sophistication or high Q-factor from masking weaknesses in the physical measurement chain.

## 5. Conclusions and Future Research

This review brings together planar microwave dielectric sensors through a design framework that links architecture, operating principle, readout, sample interface, and application. Resonant and field-confining structures enable compact, sensitive measurements, while broadband, phase-based, differential, SIW/SISL, and microfluidic approaches each address distinct needs in dynamic range, robustness, integration, and sample handling. The main point is that sensitivity or Q-factor alone does not tell the full story. Practical performance has to be judged together with calibration, uncertainty, fabrication variation, environmental stability, long-term drift, and cost.

A number of gaps remain in the literature. Definitions and reporting practices remain inconsistent for sensitivity, resolution, detection limit, accuracy, and uncertainty, making rigorous comparisons across studies difficult. Multi-laboratory validation, between-batch statistics, openly available benchmark datasets, and long-duration measurements are still relatively rare. The transferability of calibration and machine learning models across sensor devices, material families, and operating conditions is also not yet well understood.

Several technical issues remain unresolved. These include separating sample loss from substrate, conductor, radiation, and fixture losses; keeping sample geometry and temperature under control; managing tolerances in narrow-gap and multilayer fabrication; achieving stable, low-cost readout without losing traceability; and preserving calibration under aging, contamination, cleaning, thermal cycling, and humidity. Rather than optimizing only for simulated sensitivity, high-Q and high-field-confinement designs should be made less vulnerable to these non-idealities.

There are also clear opportunities for future work. Promising directions include differential and self-referenced architectures, uncertainty-aware extraction, panel-level test coupons and digital calibration records, replaceable or disposable sensing interfaces, multimodal temperature and humidity compensation, and physics-informed or transfer-learning models that explicitly account for device and environmental variation. Portable fixed-frequency electronics can also be paired with reference standards and self-test functions to support traceable field measurements.

Future work should place greater emphasis on standardized validation protocols, reference materials, shared benchmark problems, cross-device and cross-laboratory studies, lifecycle and accelerated-aging tests, tolerance-aware design with yield in mind, and clearer reporting of edge-computing cost and model uncertainty. Extending these approaches to millimeter-wave and terahertz frequencies is most valuable when it is matched with manufacturable fixtures, calibrated instrumentation, and clear application-specific advantages. Progress along these directions will help move planar microwave dielectric sensing from high-performance prototypes toward measurement systems that are reliable, scalable, and practical for industrial, biomedical, microwave-processing, and microwave wireless power transfer applications.

## Figures and Tables

**Table 1:** Comparison of resonant and non-resonant planar microwave sensors.

Ref.	Author (Year)	Measurement Method/Structure	Material Under Test (MUT)	Frequency (GHz)	Accuracy/Performance
[29]	Gou et al. (2023)	Oblique aperture ridge waveguide	Methanol/liquids	2.45	Improved sensitivity via field focusing
[49]	Wu & Zhao (2023)	Differential Magnetic-LC (MLC)	Solids (perm. and thickness)	2.38	2.49% permittivity error
[93]	Liao et al. (2021)	SIW terminal loading structure	Dielectric materials	2.45/5.8	High accuracy for ε′ and ε″
[94]	Ma et al. (2021)	Substrate-integrated suspended line	Aqueous solutions	1.48/2.54	Q-factor: 3500; 4% sensitivity
[95]	Haq & Koziel (2022)	CSRR (inverse modeling)	Industrial materials	Microwave	<3.8% max relative error
[96]	Buragohain et al. (2021)	Low-cost CSRR-based sensor	Liquid samples	Microwave	Effective for liquid characterization
[97]	Liu & Pu (2008)	Microstrip with slotted ground plane	Liquid complex permittivity	Microwave	Enhanced interaction with liquids
[92]	Liu & Tong (2015)	Substrate-integrated waveguide (SIW)	Liquids	5.85 (C-band)	Compact and integrated design
[98]	Ye et al. (2020)	Dual-mode microwave resonator	Liquid chromatography	Microwave	Targeted for chemical monitoring
[99]	Navaei et al. (2023)	Contact and free-label resonance	Aqueous solutions	Microwave	High detection accuracy
[100]	Alharbi et al. (2023)	Stepped Impedance Resonators	Magnetodielectric materials	Microwave	Multi-parameter characterization
[101]	Han et al. (2022)	Complementary Curved Ring (CCRR)	Solid materials	3.49	High field concentration
[102]	Navaei & Rezaei (2024)	Chandelier form microwave sensor	Fluidics	Microwave	High sensitivity design
[103]	Kazemi et al. (2021)	High-resolution reflective sensor	Vanadium electrolyte	5.3–6.3	Phase feature improves linearity
[104]	Sabzevari et al. (2023)	UWB Impulse Radar (GA-based)	Tunnel/Buried objects	3.1–10.6	14% max permittivity error
[105]	Samad et al. (2020)	Rectangular DS-CSRR array	PTFE, Quartz, FR4	7.01	<1.36% max permittivity error
[106]	Han et al. (2024)	CSRR Metamaterial sensor	Solids and liquids	1.53–2.45	102.2 MHz/εr resolution
[107]	García et al. (2024)	Differential SIW with CSRR	Liquid characterization	6.69–6.81	Sensitivity: 12.24 MHz/Δε_r_′
[108]	Wu & Zhao (2023)	Differential CSRs (On-chip)	Liquid samples	THz/MW	Temperature compensated
[51]	Wu et al. (2024)	Reflective-mode phase variation	Dielectric materials	2.5	1.745% maximum error for ε_r_′
[109]	Ding et al. (2023)	Epsilon-near-zero (ENZ)	Liquid crystals/powders	1.9	<3% permittivity error

**Table 2:** Comparison of microfluidic planar microwave sensors.

Ref.	Author (Year)	Measurement Method/Structure	Material Under Test (MUT)	Frequency (GHz)	Accuracy/Performance
[79]	Wu et al. (2021)	Modified CELC and SRR structures	Liquid samples	1.2/2.4	Sensitivity: 20.525 MHz/εr
[80]	Prakash et al. (2022)	Grooved CSRR-based sensor	Liquid chemicals (ethanol)	1.48/2.54	Q-factor: 3500; Sensitivity: 4%
[83]	Ye et al. (2022)	Improved SRR microfluidic sensor	Ethanol/water mixtures	1.0–3.0	High sensitivity for microliter volumes
[84]	Nguyen et al. (2023)	Filter-based microfluidic system	Liquid permittivity	2.0–6.0	Phase/attenuation based; better accuracy
[85]	Song et al. (2023)	Contactless antenna-sensor	Ethanol/methanol mixtures	2.11	Real permittivity error < 3.50%
[86]	Yasin et al. (2023)	SIW filter with capillary tube	Microliter liquid samples	4.8–5.3	Integrated single-board; 6.8% accuracy
[50]	Gan et al. (2020)	Differential MCSRR structure	Ethanol/water solutions	2.4	Suppresses environmental interference
[111]	Jang et al. (2021)	Interferometric resonator system	Glucose solutions	1.45/4.9	High resolution for glucose monitoring
[120]	Su et al. (2018)	Splitter/combiner with SRRs	Crude oil/Oil samples	2.2–2.6	Accurate complex permittivity estimation
[121]	Abdolrazzaghi (2018)	Metamaterial coupling sensor	Liquid sensing	2.5	Strongly enhanced detection sensitivity
[118]	Wu & Zhao (2022)	SRR-based sensor array	Organic tissues	Microwave	Multi-point array-based tissue analysis

**Table 3:** Design-oriented comparison of planar microwave dielectric-sensor approaches.

Approach	Preferred Use	Principal Strengths	Dominant Trade-Offs and Controls
Microstrip/CPW resonator	Low-cost laboratory or portable sensing; moderate sample volumes	Simple PCB fabrication; compact; direct frequency/Q readout	Open fields increase environmental sensitivity; connector and placement repeatability; moderate Q
SRR/CSRR/DGS	Small-volume samples; relative, threshold, or concentration sensing	Strong local field confinement; compact footprint; high sensitivity	Narrow gaps/slots are fabrication-sensitive; limited dynamic range; strong dependence on sample position
SIW/SISL	Higher-accuracy resonant characterization under controlled fabrication	High Q; shielding; reduced radiation loss; good integration	Via and layer alignment; larger footprint; multilayer cost; substrate-loss dependence
Interferometric/phase/differential	Detection of small changes under common-mode environmental drift	High fixed-frequency sensitivity; drift rejection; potential low-cost readout	Channel matching and phase noise; operating-point control; reference-channel aging
Non-resonant/broadband line	Dispersion analysis and simultaneous multi-parameter extraction	Broad frequency information; wider material range	Lower local sensitivity; stronger de-embedding and inversion burden; more data and calibration standards
Microfluidic-integrated	Biological/chemical liquids with limited sample volume	Controlled sample delivery; repeatable sensing location; disposable options	Bubbles, evaporation, channel tolerance, residue, polymer swelling, and temperature coupling

**Table 4:** Major uncertainty sources and recommended reproducibility practices.

Error Source	Typical Effect	Mitigation	Minimum Reporting
VNA and calibration residuals	Baseline/phase drift; biased S-parameters	Warm-up; suitable SOLT/TRL or de-embedding; pre- and post-reference check	Instrument, calibration method/plane, IF bandwidth, power, sweep settings, interval
Connectors, cables, and launches	Mate–de-mate variation; cable-bend phase shift; contact loss	Controlled torque; strain relief/semi-rigid cable; fixed launches; repeated connection test	Connector type, torque/procedure, number of reconnections, connection repeatability
Fixture and sample placement	Resonance shift unrelated to permittivity; parasitic coupling	Mechanical registration; fixed pressure/standoff; reference fixture; dimensional inspection	Holder geometry, pressure/standoff, alignment tolerance, positioning repetitions
Sample geometry and handling	Volume/thickness/air-gap bias; bubbles; evaporation	Metered volume; thickness measurement; bubble inspection; sealed channel; timed protocol	Volume/thickness uncertainty, loading sequence, replicate preparation, channel material
Temperature and humidity	Drift of MUT and substrate permittivity/loss	Environmental enclosure; equilibration; temperature reference; differential compensation	Temperature/humidity and stability, equilibration time, compensation method
Fabrication variation	Device-to-device frequency shift and calibration-curve change	Batch coupons; dimensional metrology; per-batch/device calibration; tolerant geometry	Board lot, substrate data, measured dimensions, between-device/batch statistics
Extraction/model uncertainty	Overfit calibration and poor extrapolation.	Independent test materials; uncertainty propagation; physics-based constraints	Calibration range, model form, residuals, blind-test error, confidence/uncertainty
Aging and contamination	Slow baseline drift; Q degradation; reference mismatch	Cleaning protocol; sealed/reference structures; drift checks; scheduled recalibration	Age/cycles, storage, cleaning, drift rate, recalibration criterion

## Data Availability

No new data were created or analyzed in this review article. All information discussed in this review was obtained from previously published studies cited in references. Data sharing is not applicable to this review.

## Abbreviations

The following abbreviations are used in this manuscript:

AI	Artificial intelligence
ANN	Artificial neural network
CPW	Coplanar waveguide
CMA-ES	Covariance Matrix Adaptation Evolution Strategy
CSRR	Complementary split-ring resonator
DBSCAN	Density-Based Spatial Clustering of Applications with Noise
DGS	Defected ground structure
FDS	Frequency-domain dielectric spectroscopy
GA	Genetic algorithm
IDC	Interdigital capacitor
ML	Machine learning
MLA	Machine-learning-aided
MLP	Multilayer perceptron
MIMO	Multiple-input multiple-output
MUT	Material under test
NDE	Nondestructive evaluation
PCB	Printed circuit board
RFID	Radio-frequency identification
SAR	Synthetic aperture radar
SIW	Substrate-integrated waveguide
SRR	Split-ring resonator
SVM	Support vector machine
TDR	Time-domain reflectometry
TDS	Time-domain dielectric spectroscopy
THz	Terahertz
UWB	Ultra-wideband
VNA	Vector network analyzer
